# miRNA-Mediated Regulation of γ-Globin to β-Globin Switching: Therapeutic Potential in β-Hemoglobinopathies

**DOI:** 10.3390/ijms27031203

**Published:** 2026-01-25

**Authors:** Daniah Alotaibi, Falak Aldagdog, Sajidah Alramadhan, Basmah Almuhaidib, Nada Asiri, Leena Almodhi, Manar Alshabaan, Razan Alborhan, Chittibabu Vatte, Shamim Shaikh Mohiuddin, Amein K. Alali, Alawi Habara

**Affiliations:** 1 College of Medicine, Imam Abdulrahman Bin Faisal University, P.O. Box 1982, Dammam 31441, Saudi Arabia; 2230002629@iau.edu.sa (D.A.); 2230004381@iau.edu.sa (F.A.); 2230002317@iau.edu.sa (S.A.); 2230002030@iau.edu.sa (B.A.); 2230000427@iau.edu.sa (N.A.); 2230006356@iau.edu.sa (L.A.); 2220002378@iau.edu.sa (M.A.); 2 School of Medicine, University College Dublin, Belfield, Dublin 4, Ireland; razan.alborhan@ucdconnect.ie; 3 Department of Biochemistry, College of Medicine, Imam Abdulrahman Bin Faisal University, P.O. Box 1982, Dammam 31441, Saudi Arabia; cbvatte@iau.edu.sa (C.V.); smohiuddin@iau.edu.sa (S.S.M.); aalali@iau.edu.sa (A.K.A.)

**Keywords:** β-globin gene cluster regulation, hemoglobin switch regulation, miRNA, sickle cell disease, β-thalassemia, nanoparticles

## Abstract

Erythropoiesis is a tightly regulated developmental process that requires the switch from fetal hemoglobin (HbF) to adult hemoglobin (HbA). In β-hemoglobinpathies such as SCD and β-thalassemia, disease severity is influenced by the fetal-to-adult hemoglobin switch because persistence or induction of HbF will ameliorate the clinical manifestations. miRNAs play an essential role in regulating this switch by modulating the expression levels of key transcription factors, such as BCL11A, KLF1, and MYB, which repress γ-globin expression. Multiple miRNAs have been identified as potential modulators of the hemoglobin switch, including miR-144, miR-486, miR-26b, and miR-15a. The molecular interactions between miRNA and γ-to β-globin switch have the potential for new therapeutic interventions that aim to reactivate HbF expression to ameliorate β-hemoglobinopathies such as SCD and β-thalassemia. In this review, the latest advancements in miRNA-mediated regulation of Hb switching and nanoparticle-based strategies for miRNA delivery are explored.

## 1. Introduction

β-hemoglobinopathies are genetic disorders affecting the structure or production of hemoglobin (Hb), the oxygen-carrying protein in red blood cells. These disorders arise due to mutations in the *HBB* or *HBA* genes, leading to abnormal Hb variants or reduced Hb synthesis. Sickle cell disease (SCD) is an inherited hematologic disorder caused by a single-nucleotide mutation in the *HBB* gene, which encodes the β-globin subunit of hemoglobin (*HBB*: c.20A > T) [1,2,3,4,5]. This mutation leads to the substitution of glutamic acid with valine at position six of the β-globin chain (Glu6Val), resulting in the production of sickle hemoglobin (HbS) instead of normal adult hemoglobin (HbA). The disease typically follows an autosomal recessive pattern, where individuals with two mutated copies of *HBB* (β^s^/β^s^ genotype) develop sickle cell anemia (SCA), whereas those with one mutated and one normal allele (β^S^/β^A^ genotype) are carriers, commonly referred to as having sickle cell trait, and usually do not exhibit disease symptoms [1,2,3,4,6]. Nevertheless, some reports described sickle cell disease caused by a cis “double” mutation on the same *HBB* allele, for example, HbS-Oman, Hb-Jamaica Plain, and Hb-São Paulo [7,8,9], which are rare and typically have an autosomal dominant inheritance pattern.

At the molecular level, HbS undergoes polymerization under hypoxic conditions, which causes red blood cells (RBCs) to become rigid and deformed into the characteristic sickle shape [1,2,3,4,10]. These misshapen RBCs exhibit reduced deformability and increased adhesiveness, which promotes vascular occlusion and ischemic injury in tissues and organs [1,2,3,4,10]. The mechanical fragility of sickled cells leads to chronic hemolysis, reducing RBC lifespan and contributing to anemia and compensatory erythropoiesis in the bone marrow. The repetitive cycles of vaso-occlusion and hemolysis drive a state of systemic inflammation, endothelial activation, and oxidative stress, which exacerbate the disease burden and contribute to progressive organ damage [11,12]. Additionally, recurrent microvascular occlusion followed by reperfusion results in ischemia–reperfusion (I/R) injury, which drives endothelial activation, inflammation, and multi-organ pathology [11,12,13,14,15]. Reoxygenation converts xanthine oxidoreductase to xanthine oxidase, generating reactive oxygen species and promoting leukocyte recruitment and vascular dysfunction. I/R injury extends beyond the occluded microvascular bed: the lungs are the principal site of damage, with additional involvement of the liver and heart [13,14]. In line with these considerations, nationwide inpatient data from 2020 indicate that SCD patients presenting with acute coronary syndrome are younger [6]. While in-hospital mortality and time to percutaneous coronary intervention (PCI) mirror those of non-SCD patients, SCD is associated with a markedly increased risk of coronary artery dissection during PCI, warranting careful procedural planning [6].

Similarly, β-thalassemia is an autosomal recessive hemoglobinopathy caused by diverse mutations in the *HBB* gene, resulting in reduced (β^+^) or absent (β^0^) synthesis of β-globin chains [16,17]. Rare autosomal-dominant forms also occur, typically due to unstable β-globin variants; for example, hemoglobin Dieppe (*HBB*:c.383A > G; β127Gln → Arg), which produces a dominant β-thalassemia phenotype through globin instability [18]. This deficiency leads to an imbalance between α- and β-globin chains, with excess α-globin chains precipitating within erythroid precursors and mature red blood cells. Such precipitation causes ineffective erythropoiesis, apoptosis of erythroid precursors, severe hemolytic anemia, and iron overload secondary to increased intestinal iron absorption and repeated blood transfusions in severe cases [16,17]. Individuals with severe forms of β-thalassemia are often transfusion-dependent, requiring lifelong supportive care, and experience substantial morbidity from chronic anemia, ineffective erythropoiesis, extramedullary hematopoiesis, and organ damage due to iron accumulation [16,17].

**Figure 1:**
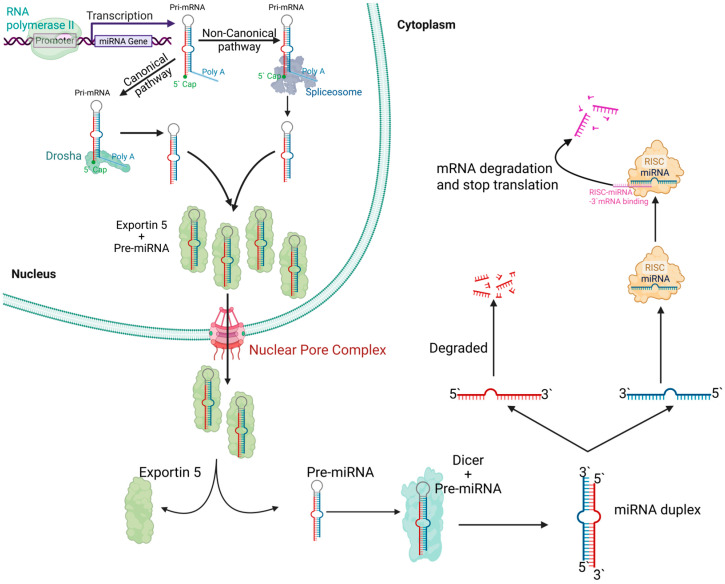
Biogenesis and function of miRNAs. miRNA genes are transcribed by RNA polymerase II into primary transcripts (pri-miRNAs), which undergo processing through either the canonical pathway (the Drosha complex in the nucleus) or non-canonical pathways (the spliceosome). The resulting precursor miRNAs (pre-miRNAs) are exported to the cytoplasm by Exportin-5 through the nuclear pore complex. In the cytoplasm, Dicer further processes pre-miRNAs into double-stranded miRNA duplexes. One strand of the duplex (the guide strand) is loaded into the RNA-induced silencing complex (RISC) containing Argonaute proteins (AGO2). Mature RISC–miRNA complexes regulate target mRNAs by promoting translation suppression or mRNA cleavage and degradation, while the passenger strand is degraded.

miRNAs are small, non-coding RNA molecules, about 20 to 23 nucleotides long [19,20], that regulate gene expression post-transcriptionally by binding to target mRNAs to inhibit translation or trigger degradation [19,20]. They are initially transcribed as long primary transcripts, pri-miRNAs, that can be produced by either RNA polymerase II, which generates capped and polyadenylated transcripts, or RNA polymerase III, which uses distinct promoter and termination elements for transcription [19,20]. There are two main pathways for miRNA processing: the canonical pathway and the non-canonical pathway (Figure 1). In the canonical pathway, pri-miRNAs fold into characteristic hairpin structures and are processed in the nucleus by the Drosha-DGCR8 microprocessor complex to produce precursor miRNAs (pre-miRNAs) [19,20]. These pre-miRNAs are exported to the cytoplasm, where the RNase Dicer cleaves them into a miRNA duplex, one strand of which is then loaded into the RNA-induced silencing complex (RISC) to mediate gene silencing [19,20]. In contrast, non-canonical pathways bypass one or more of these standard processing steps by employing alternative mechanisms, such as splicing-dependent formation of pre-miRNA hairpins or direct Ago2-mediated cleavage, to generate mature miRNAs that are also incorporated into RISC. Despite these differences in biogenesis, both pathways converge on producing functional miRNAs that regulate gene expression via translational repression or mRNA degradation [19,20].

In β-hemoglobinopathies, precise control of globin gene expression is essential for balancing HbF and HbA production. Recent research has demonstrated that epigenetic mechanisms and specific miRNAs are crucial in regulating the developmental switch between γ and β-globin. Certain miRNAs target key transcriptional repressors, such as BCL11A and MYB, as well as epigenetic modifiers like DNA methyltransferases, thereby relieving the suppression of γ-globin expression and enhancing HbF production [21,22,23,24,25]. Moreover, distinct miRNA expression profiles in erythroid cells not only provide insight into the molecular pathways governing hemoglobin switching but also serve as sensitive biomarkers for disease severity in conditions like sickle cell disease. These findings have significant clinical implications, as miRNA-based therapeutic strategies that elevate HbF levels can potentially ameliorate clinical symptoms and improve outcomes in patients with β-hemoglobinopathies [26,27]. Additionally, miRNAs are involved in a broader circRNA–miRNA–mRNA network [25,28]; however, we do not elaborate on miRNA-circRNA interactions here and instead center this review on how miRNAs modulate the γ-to β-globin transition alongside established transcription factors.

## 2. miRNA-Mediated Upregulation of HbF Expression

**Figure 2:**
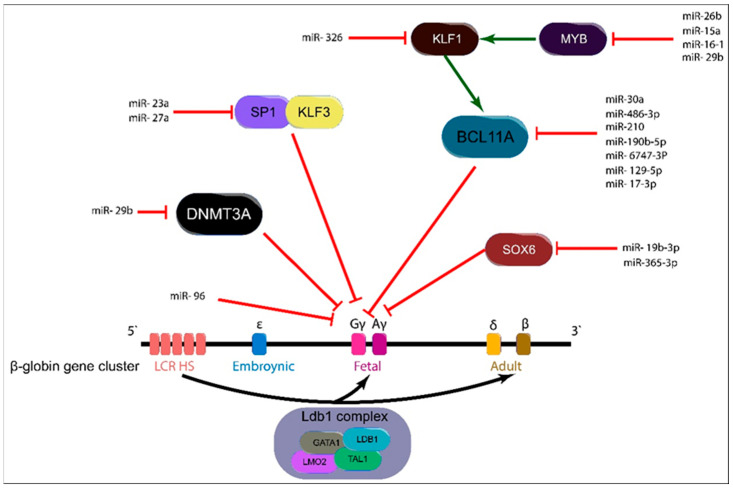
miRNA-mediated regulation of fetal hemoglobin expression. Multiple transcription factors and epigenetic regulators converge to repress γ-globin expression within the β-globin gene cluster. Key repressors include BCL11A, MYB, KLF1, SOX6, SP1, KLF3, and DNMT3A, which act directly or indirectly to silence the γ-globin promoters. Specific miRNAs target these repressors, thereby relieving inhibition and promoting HbF production. For example, miR-30a, miR-210, miR-486-3p, miR-190b-5p, miR-6747-3p, miR-129-5p, and miR-17-3p inhibit BCL11A; miR-26b, miR-15a/16-1, and miR-29b target MYB; miR-326 suppresses KLF1; miR-365-3p and miR-19b-3p repress SOX6; miR-23a and miR-27a target SP1 and KLF3; and miR-29b inhibits DNMT3A, leading to epigenetic derepression of γ-globin. Additionally, miR-96 directly modulates γ-globin mRNA translation. Collectively, these interactions shift the balance toward γ-globin expression, enhancing HbF synthesis and ameliorating the pathophysiology of β-hemoglobinopathies such as sickle cell disease and β-thalassemia.

In recent years, considerable attention has focused on the role of miRNAs in upregulating HbF expression by modulating key transcription factors that normally repress γ-globin gene expression. Transcription factors such as BCL11A, SOX6, MYB, KLF1, and the SP1-KLF3 axis are critical regulators of the HbF-to-HbA switch; under normal conditions, they suppress HbF levels during adult erythropoiesis [29,30,31]. However, specific miRNAs have been identified that target these repressors, thereby relieving their inhibitory effects and promoting HbF production [31]. We review the current evidence on how miRNAs that target *BCL11A*, *SOX6*, *MYB*, *KLF1*, *SP1*-, and *KLF3* contribute to the reactivation of γ-globin expression, highlighting their potential as therapeutic agents for β-hemoglobinopathies such as sickle cell disease and β-thalassemia (Figure 2).

### 2.1. miRNA-BCL11A Axis

The *BCL11A* gene, located on chromosome 2p16.1 and comprising four exons and three introns, encodes a zinc-finger protein that functions as a transcriptional repressor [29,30]. BCL11A is the central transcription factor in the formation of the BCL11A-MBD2-NuRD complex, which represses γ-globin formation [2,32,33,34,35]. The extra-long isoform, BCL11A-XL, is the predominant variant in adult erythroblasts [33,36]. The BCL11A-XL isoform C-terminal zinc fingers (ZnF4-6) bind the *HBG* promoters, leading to silencing of γ-globin production [33,36]. Reduction in the BCL11A-XL isoform or disruption of ZnF4-6 derepresses γ-globin and increases HbF [33,36]. The shorter BCL11A isoforms, predominant in fetal and primitive erythroid cells, lack the XL-specific ZnF4-6 domain and therefore lack the repressive capacity to silence the *HBG* promoters [36].

Several miRNAs converge on *BCL11A* to modulate HbF levels. In β-thalassemia intermedia, miR-30a-5p binds the 3′ untranslated region of *BCL11A* mRNA, reducing its stability and translation and thereby increasing HbF; elevated miR-30a in erythroid precursors correlates strongly with higher HbF, underscoring the therapeutic potential of the miR-30a-*BCL11A* interaction [23].

Convergent evidence also supports a miR-210–BCL11A pathway. In CD34^+^-derived erythroid precursors, transfection with miR-210 induces a dose-dependent reduction in the *BCL11A-XL* isoform at mRNA and protein levels, with a corresponding rise in γ-globin expression [22,37]. Importantly, flow cytometry analysis shows that key erythroid markers, such as CD71 and CD235a, remain unchanged after miR-210 treatment, indicating that the modulation is specific to the globin gene switch rather than affecting overall erythroid differentiation [37]. These findings underscore the therapeutic potential of targeting miR-210 to elevate HbF levels and improve clinical outcomes in β-hemoglobinopathies like β-thalassemia.

Next, miR-486-3p regulates HbF production by directly modulating *BCL11A-XL* in human erythroid cells. In primary human CD34^+^-derived erythroid cell culture, miR-486-3p is minimally expressed in early progenitors but becomes significantly upregulated during erythroid differentiation [38]. Overexpression of miR-486-3p results in a ~40% reduction in BCL11A protein levels and a concomitant increase in γ-globin mRNA and protein [38]. Conversely, inhibition of miR-486-3p leads to higher BCL11A levels and reduced γ-globin expression. Furthermore, erythroid cells from β-thalassemia patients show significantly higher miR-486-3p expression, which correlates with increased HbF synthesis. These findings indicate that miR-486-3p is a potent post-transcriptional mechanism controlling HbF levels and a promising therapeutic target for β-hemoglobinopathies [38]. Additionally, miR-486-3p has a modest prediction value to differentiate patients with STEMI from those with stable ischemic heart disease [39]. miR-486-3p dual relevance to globin regulation and coronary events positions it as a plausible diagnostic and prognostic tool across β-hemoglobinopathies who suffer from acute coronary syndrome.

Another relevant miRNA, miR-190b-5p, significantly upregulates HbF production. In pediatric β-thalassemia, miR-190b-5p is markedly upregulated in peripheral blood and shows a significant inverse correlation with *BCL11A* mRNA levels while positively correlating with increased HbF. Luciferase reporter assays confirmed that miR-190b-5p directly binds to the 3′-UTR of *BCL11A*, leading to its downregulation and consequent enhancement of HbF production. Additionally, ROC analysis indicates that hsa-miR-190b-5p has a diagnostic value comparable to HbA2, and combining both markers further improves diagnostic accuracy. These findings underscore the clinical relevance of miR-190b-5p as a potential biomarker and therapeutic target for modulating HbF levels in pediatric β-thalassemia [40].

Also relevant is miR-6747-3p; it significantly upregulates HbF production in β-thalassemia. miR-6747-3p was found to be markedly upregulated in patients with β-thalassemia major relative to healthy controls, with its expression positively correlating with increased HbF levels. Functional assays in HUDEP-2 and K562 demonstrated that overexpression of miR-6747-3p not only impairs cell proliferation and promotes apoptosis but also accelerates erythroid differentiation, leading to enhanced γ-globin expression. Mechanistically, miR-6747-3p directly binds to the 546–552 nucleotide region of the BCL11A mRNA 3′-UTR, thereby downregulating this key transcriptional repressor of fetal hemoglobin. These findings underscore the potential of targeting the miR-6747-3p-*BCL11A* axis as a novel therapeutic strategy to induce HbF production in β-thalassemia [41].

Another miRNA, miR-129-5p, is upregulated in β-thalassemia and associated with higher HbF [42]. Overexpression of miR-129-5p in erythroid cell models, K562 and HUDEP-2, demonstrates that miR-129-5p directly targets the BCL11A 3′-UTR, leading to BCL11A downregulation and, in turn, inducing HbF production. Additionally, miR-129-5p promotes erythroid maturation [42]. miR-129-5p level was also found to be inversely associated with serum alanine aminotransferase (ALT) and aspartate aminotransferase (AST) [42], supporting its use as a noninvasive marker of hepatocellular injury. Taken together, these data support evaluating miR-129-5p both as a therapeutic target for HbF modulation and as a biomarker in β-hemoglobinopathies, including for monitoring liver damage.

miR-17-3p is upregulated in peripheral blood from β-thalassemia patients and positively correlates with HbF; it also shows good discrimination between patients and controls, AUC ≈ 0.88, and between high vs. low HbF cases, AUC ≈ 0.93 [43]. It directly targets BCL11A 3′UTR, lowering BCL11A mRNA and protein, thereby increasing γ-globin in K562 cells [43]. Overall, miR-17-3p is a promising candidate for HbF-inducing therapy and a potential biomarker for monitoring β-thalassemia.

### 2.2. miRNA-MYB-KLF1-BCL11A Axis

The *MYB*-*KLF1*-*BCL11A* axis is an upstream pathway that enforces the switch from HbF to HbA [44,45]. MYB indirectly regulates γ-globin by activating KLF1 [3], which then silences γ-globin by upregulating the repressor BCL11A [3,44,46] (Figure 2).

Hydroxyurea involves this axis via miR-26b; hydroxyurea elevates miR-26b, which binds to *MYB* mRNA at its 3′-UTR and promotes RISC-mediated decay, lowering MYB protein level, which in turn lowers KLF1 and BCL11A; γ-globin is consequently derepressed, and HbF increases [47]. Consistently, enforced miR-26b induces γ-globin in K562 erythroid cells [47,48].

Another miRNA, miR-15a/16-1, acts similarly to regulate HbF expression. Overexpression of miR-15a and miR-16-1 in primary CD34^+^ cells and K562 cells using lentiviral vectors robustly increased γ-globin and embryonic ε-globin levels. Luciferase reporter assays confirmed that these miRNAs directly target the *MYB* 3′-UTR, and Western blot analyses demonstrated a reduction in MYB protein [49]. Complementary shRNA-mediated knockdown of MYB similarly elevated HbF, linking miR-15a/16-1 suppression of MYB and the consequent decrease in BCL11A activation to enhanced γ-globin synthesis [49].

Another miRNA, miR-29b, inhibits the de novo synthesis of the DNMT enzyme, specifically DNMT3A, which leads to the upregulation of HbF expression in primary human CD34^+^ derived erythroid cell culture [50]. Further investigation of miR-29b revealed that it also inhibits *MYB* in KU812 leukemia cells, primary human CD34^+^ derived erythroid cells, and in the Townes SCD mouse model [51].

Some miRNAs work through *KLF1* directly; an example of this is miR-326, which targets the *KLF1* 3′-UTR, lowering KLF1 protein; because KLF1 activates BCL11A, this downshifts BCL11A and can derepress γ-globin and increase HbF [24] (Figure 2). In K562 cells, miR-326 overexpression increased HbF. In primary CD34^+^ progenitors, miR-326 reduced KLF1 protein and *BCL11A* mRNA but did not significantly change γ- or β-globin transcripts; however, it reduced erythroid maturation (lower glycophorin-A), indicating context-dependent effects. In β-thalassemia major reticulocytes, higher miR-326 was positively correlated with HbF [24], consistent with a role in stress erythropoiesis and supporting its evaluation as a therapeutic target and biomarker.

### 2.3. miRNA-SOX6-BCL11A Axis

The *SOX6* gene is located on chromosome 11p15.2 [52]. SOX6 is an HMG-box transcription factor that collaborates with BCL11A to enforce γ-globin silencing during definitive erythropoiesis [52,53]. In adult human erythroid progenitors, SOX6 and BCL11A are coexpressed, physically interact, co-occupy the β-globin cluster, and act together to repress γ-globin transcription [54]. Consistent with a role in the mature erythroid program, SOX6 overexpression accelerates human erythroid differentiation, highlighting its importance in late erythropoiesis [54]. Conversely, lowering SOX6 can derepress γ-globin. Lentiviral RNAi knockdown increased γ-globin in K562 cells and in erythroblasts derived from normal donors and from patients with β-thalassemia major without impairing maturation, and CRISPR/Cas9 disruption of the SOX6 binding domain in K562 cells raised γ-globin mRNA levels [54,55,56].

In a mouse fetal liver model, complete loss of BCL11A led to significant upregulation of miR-365-3p, which directly targets the 3′ UTR of *SOX6*, reducing its expression and derepressing embryonic and fetal globin genes [57]. In human HUDEP-2 erythroid cells, overexpression of miR-365-3p similarly decreased SOX6 levels at both the mRNA and protein levels, resulting in a marked increase in fetal globin expression [57]. Additionally, luciferase reporter assays in HeLa cells confirmed that miR-365-3p directly binds to a conserved seed region within the *SOX6* 3′ UTR. These results delineate an evolutionarily conserved regulatory pathway involving BCL11A, miR-365-3p, and SOX6 that modulates hemoglobin switching and may offer new therapeutic avenues for β-hemoglobinopathies [57].

Another miRNA involved in the SOX6 pathway is miR-19b-3p. It modulates HbF levels in β-thalassemia. miR-19b-3p was significantly up-regulated in β-thalassemia carriers with high HbF, and this upregulation correlates with a marked downregulation of SOX6 expression. Functional assays, including RNA immunoprecipitation and dual-luciferase reporter experiments, confirm that miR-19b-3p directly binds to the 3′-UTR of *SOX6*, leading to its post-transcriptional repression. This interaction relieves the inhibitory effect of SOX6 on fetal globin gene expression, thereby contributing to HbF induction [58].

### 2.4. miRNA-SP1/KLF3 Axis

Specificity protein 1 (Sp1) is a transcription factor. It binds GC/GT boxes across the β-globin locus control region (LCR) and the ε/γ/β promoters and represses β-like globin gene expression [59]. In undifferentiated cells, Sp1 occupancy helps recruit HDAC1, keeping histones less acetylated and chromatin closed; upon differentiation, Sp1 becomes phosphorylated and dissociates from HS2–HS4 and the γ-globin promoter, allowing FKLF2 to recruit the histone acetyltransferase and increase histone acetylation, thereby enabling β-like globin gene activation [59].

miR-23a and miR-27a promote the expression of globin genes within the β-globin locus by directly repressing the transcriptional repressors SP1 and KLF3 [60]. As these miRNAs increase, SP1/KLF3 occupancy across the locus decreases, relieving repression and increasing ε- and γ-globin in K562 cells, whereas in primary CD34^+^ erythroid cultures the dominant effect is β-globin induction [60]. Because KLF3 also binds and represses the promoter of the miR-23a/27a/24-2 cluster, miR-23a further lowers KLF3, creating a positive feedback loop that amplifies both miRNA levels and globin gene expression [60]. These differing responses reflect the developmental stage-specific programming of the β-globin locus; K562 cells exhibit an embryonic/fetal-like (ε/γ-biased) phenotype, whereas CD34^+^ cultures mature toward the adult program (β-predominant).

### 2.5. miR-96-γ-Globin Axis

The miR-96-γ-globin axis operates through direct post-transcriptional repression, modulating HbF production. In adult erythroid reticulocytes, where HbF levels are low, a substantial portion of γ-globin mRNA is sequestered within AGO2-containing RNA-induced silencing complexes, a phenomenon that is markedly less pronounced in umbilical cord blood reticulocytes, which exhibit high HbF levels. Ex vivo erythropoiesis models using cord blood-derived and bone marrow-derived erythroblasts demonstrate that overexpression of miR-96 results in approximately a 50% reduction in c-globin protein expression, while knockdown of miR-96 leads to a 20% increase [61]. Moreover, luciferase reporter assays in HEK293T cells reveal that miR-96 directly binds to a highly complementary, seedless target site within the open reading frame of γ-globin mRNA. Collectively, these findings indicate that miR-96 plays a critical role in fine-tuning HbF levels by inhibiting γ-globin translation during human erythropoiesis [61]. Table 1 summarizes miRNAs and their targets.

## 3. Use of miRNA as Therapy for β-Hemoglobinopathies

RNA interference-based approaches using lentiviral vectors encoding short hairpin miRNA (shmiR) represent promising therapeutic strategies for β-hemoglobinopathies by achieving erythroid-specific knockdown of critical transcriptional repressors such as BCL11A. A double shmiR lentiviral vector that simultaneously targets BCL11A and ZNF410, two key repressors of γ-globin [69]. This dual approach achieves greater HbF induction than either single vector alone, reaching ~49% HbF compared with ~40% using BCL11A-only shmiR and lower levels with ZNF410-only shmiR [69]. In sickle cell patients, derived hematopoietic stem cells, HbF increased from ~32% with BCL11A shmiR to ~45% with the double shmiR, accompanied by reduced erythrocyte sickling and improved erythroid maturation [69].

Additionally, a novel bifunctional lentiviral vector incorporating shmiRs targeting both BCL11A and ZNF410 [69], combined with a modified anti-sickling β-globin (β^AS3^-globin) gene, demonstrated significant induction of HbF, robust anti-sickling effects, and reduced erythrocyte sickling in CD34^+^ hematopoietic stem and progenitor cells from SCD patients, as well as in the Berkeley sickle cell mouse model [70,71].

Currently, the only shmiR-based therapy to reach the clinic is BCH-BB694, an erythroid-restricted vector that silences BCL11A. In the ongoing NCT03282656 trial, treated patients have demonstrated reliable engraftment, durable BCL11A knockdown, and HbF induction ranging from ~20–41%, accompanied by clinical improvement and, importantly, no evidence of clonal dominance to date [21,72]. Collectively, these findings support the use of RNA interference-based silencing of BCL11A as a clinically effective strategy for inducing fetal hemoglobin production in sickle cell disease and related β-hemoglobinopathies.

Beyond vector-based RNA-interference strategies, systemically delivered miRNA therapeutics can induce HbF without genomic integration. In a Townes sickle cell mouse model, daily subcutaneous cholesterol-conjugated miR-29b for four weeks increased HbF level, expanded HbF-positive red cells, improved spleen pathology, and did not alter blood counts [73]. Mechanistically, the treatment reduced DNMT3A and demethylated the γ-globin promoter, consistent with HbF reactivation [73].

The presence of the erythroid-specific enhancer and transcript puts BCL11A at an advantage over other HbF transcription factors. Functional studies identify the long isoform, BCL11A-XL, as a key mediator of γ-globin silencing/HbF repression during erythroid maturation [30,74]. MYB is also regulated by erythroid-active distal enhancers in the HBS1L–MYB intergenic region that influence HbF levels [75], but MYB lacks the erythroid-specific isoform, which makes BCL11A a much more suitable candidate for miRNA targeting than MYB.

## 4. Use of Nanoparticles as a Delivery Strategy for miRNA-Based Therapies

Nanoparticles are engineered, nanoscale carriers designed to encapsulate or conjugate therapeutic cargo, such as small molecules or nucleic acids, and deliver it to specific cells or tissues [76,77,78]. Nanoparticles protect the cargo from degradation and enable targeted uptake and controlled release. Therefore, nanoparticle delivery can improve bioavailability and therapeutic efficiency while helping to limit systemic exposure and off-target effects [76,77,78].

To induce HbF safely, therapeutic nanoparticles must reach erythroid precursors in the bone-marrow niche rather than mature red cells, which are anucleate and cannot change gene expression. A practical design first biases particles toward the marrow by engaging chemokine and adhesion cues on the sinusoidal endothelium and then exploits transferrin receptor-1 (CD71) on developing erythroblasts for endocytic entry, with glycophorin A (CD235a) enhancing selective binding and retention within erythroid islands [76,77,78]. Therapeutic constructs intended to elevate HbF, such as antisense oligonucleotides directed at the *BCL11A* enhancer, miRNA modulators, or genome-editing systems, should be engineered for erythroid selectivity and formulated with endosomal-escape functionality to ensure productive cytosolic/nuclear entry at the site of action.

In practice, in vivo HbF induction remains limited by delivery, as the liver and spleen clear most nanoparticles before they reach the bone marrow. Even with homing ligands, marrow enrichment is modest. CD71 supports uptake but is not erythroid-specific, creating off-target sinks, whereas glycophorin A improves localization but does not drive internalization. After receptor-mediated endocytosis, endosomal escape is a major bottleneck. Current HbF-inducing constructs, such as *BCL11A* enhancer silencers and genome editors, are relatively large and labile, which complicates formulation and elevates safety concerns. A recent in vivo demonstration of CD117-targeted lipid nanoparticles delivering RNA directly to bone marrow hematopoietic stem and progenitor cells suggests that marrow access and receptor-mediated entry are technically achievable. However, off-target biodistribution, antibody/chemistry dependence, and rodent model constraints temper immediate translational claims [79]. Together with the high efficacy of ex vivo HSPC gene therapy [80,81], these constraints explain why in vivo nanoparticle strategies for β-hemoglobinopathies have not yet translated to the clinic. Table 2 summarizes some of the known nanoparticles targeting HSPCs.

By contrast, RBC-based nanoparticles, drug-loaded RBC carriers, RBC–membrane–coated nanoparticles, and nano-erythrosomes are optimized for prolonged intravascular residence via self-markers, such as CD47, thereby minimizing reticuloendothelial clearance [65]. They are poorly suited to HbF induction but align with adjunct management of acute vaso-occlusive crisis: long-circulating, hemocompatible carriers can deliver anti-adhesion agents that attenuate P-/E-selectin and VCAM-1–mediated interactions, antioxidants or heme-scavenging moieties that mitigate endothelial injury, deoxyribonuclease to dismantle neutrophil extracellular traps, and stimulus-responsive nitric oxide donors to improve microvascular perfusion.

## 5. Discussion

The regulation of Hb switching from HbF (α_2_γ_2_) to HbA (α_2_β_2_) represents a central therapeutic target in β-hemoglobinopathies such as SCD and β-thalassemia [1,12]. Mounting evidence suggests that miRNAs play a crucial role in regulating this process post-transcriptionally, acting through the coordinated repression of transcription factors and epigenetic modulators that normally silence γ-globin expression [21,22,23,34,40,42,47,48,49,51,61,66,71]. Among these, miRNAs targeting BCL11A are particularly well characterized and include miR-30a-5p, miR-210, miR-486-3p, miR-190b-5p, miR-6747-3p, miR-129-5p, and miR-17-3p consistently derepress γ-globin, highlighting BCL11A as the most intensively studied and therapeutically validated node [22,23,37,38,39,40,41,62,63,64,65].

Other critical pathways include the MYB–KLF1–BCL11A axis, where miRNAs such as miR-26b, miR-15a/16-1, miR-29b, and miR-326 modulate upstream regulators, and the SOX6 pathway, where miR-365-3p and miR-19b-3p relieve repression of γ-globin transcription [14,24,47,48,49,50,51,57,68]. Additional miRNAs, such as miR-23a/27a and miR-96, further expand the regulatory network, underscoring the complexity and redundancy of miRNA-mediated control of globin gene expression. Table 1 summarizes the miRNAs and their targeted axes.

SP1 is another transcription repressor for the β-globin locus. Sp1 helps keep chromatin closed in immature cells, so reducing Sp1 activity favors β-like globin gene activation during erythroid maturation. miR-23a and miR-27a boost globin expression by directly suppressing the repressors SP1 and KLF3, relieving repression across the locus; the exact globin genes induced (ε/γ vs. β) depend on the developmental state of the cells, and a positive feedback loop via KLF3 further amplifies this pro-globin effect [60].

By selectively repressing γ-globin silencers, miRNAs can increase HbF to levels known to mitigate hemoglobin polymerization, reduce sickling, and improve erythrocyte survival. Clinical observations have already confirmed that patients with naturally elevated HbF, whether due to genetic modifiers or stress erythropoiesis, exhibit milder disease phenotypes. Harnessing miRNAs to reproduce this protective state represents a promising strategy to modify the disease course. Moreover, the dual roles of certain miRNAs, such as miR-486-3p (with a prognostic value in cardiovascular disease) and miR-129-5p (as a biomarker of liver injury), highlight their potential as both therapeutic targets and diagnostic/prognostic tools.

The translation of these insights into therapy has been facilitated by advances in RNA interference and gene therapy technologies. Vector-based strategies encoding shmiRs against *BCL11A* and *ZNF410*, as well as lentiviral constructs like BCH-BB694 [69], demonstrate robust HbF induction in both preclinical models and clinical trials. Parallel approaches using chemically modified, systemically delivered miRNAs (e.g., miR-29b mimics) provide an alternative strategy that avoids permanent genomic modification while still inducing HbF. These complementary platforms illustrate the versatility of miRNA-based therapeutics and raise the possibility of tailored interventions based on patient-specific disease severity, comorbidities, or treatment accessibility.

Compared with ex vivo autologous HSC gene therapy, which requires stem cell harvesting, ex vivo manipulation, reinfusion, and conditioning regimens, such as myeloablation, that can cause substantial toxicity and limit broad applicability [79,81]. The miRNA-based therapy approach could be positioned as a more scalable, pharmacologic alternative if efficient erythroid/HSPC targeting is achieved [79]. Moreover, individualized manufacturing and specialized centers contribute to prohibitive costs and access barriers for ex vivo approaches, whereas drug-like miRNA platforms may better support standardized production and dose adjustment or repeat administration [79].

Nevertheless, several challenges remain. First, the pleiotropic nature of miRNAs means that off-target effects must be carefully evaluated, particularly given their roles in erythropoiesis, immune modulation, and other tissue-specific pathways. Second, the long-term safety and durability of HbF induction remain critical questions, especially for systemically delivered miRNAs. Third, inter-patient variability in miRNA expression profiles suggests that personalized strategies may be required for optimal benefit. Addressing these challenges will require integrated approaches combining molecular profiling, preclinical validation, and carefully designed clinical studies [86].

## 6. Conclusions

miRNAs have emerged as central regulators of globin gene expression, orchestrating a network of transcriptional and epigenetic pathways that control the HbF-to-HbA switch. Experimental and clinical data consistently demonstrate that targeting repressors such as BCL11A, MYB, KLF1, and SOX6 through specific miRNAs can effectively reactivate γ-globin expression and increase HbF levels. These findings not only deepen our understanding of hemoglobin regulation but also provide a rational framework for the development of miRNA-based therapeutics.

As the field advances, miRNA-directed therapies, whether via viral vectors or nanoparticles, hold significant promise for transforming the management of β-hemoglobinopathies. By restoring HbF, such therapies can address the root pathophysiology of SCD and β-thalassemia rather than merely alleviating symptoms. In addition, the diagnostic and prognostic potential of miRNAs as biomarkers further enhances their clinical utility. Future research should focus on optimizing delivery strategies, ensuring long-term safety, and integrating miRNA-based approaches with existing treatments such as hydroxyurea and gene editing. Ultimately, miRNA therapeutics represent a powerful and innovative avenue toward disease-modifying and potentially curative interventions for β-hemoglobinopathies.

## Figures and Tables

**Table 1:** List of miRNAs involved in HbF production.

miRNA-BCL11A Axis					
miRNA	Sequence Location	Predicted Mechanism	Study Model	Comment/Association with Heart or Liver Injuries	References
miR-30a-5p	Chr6: 71403551—71403621	Reduced the expression of BCL11A	Primary human CD34+ derived erythroid cell culture obtained from β-thalassemia intermediate patients	miR-30a-5p is significantly elevated in patients with LV dysfunction after acute myocardial infarction. Potentially hepatoprotective.	[23,62,63]
miR-210-3p	chr11: 568089–568198	Bind to BCL11A-XL	Primary human CD34+ derived erythroid cell culture obtained from β-thalassemia patients K562 cell culture	The modulation is specific to the globin gene switch rather than affecting overall erythroid differentiation. In hepatic cells, miR-210-3p mediates ischemia-reperfusion cell injury. miR-210-3p is associated with heart failure and adverse ventricular remodeling.	[22,37,64,65]
miR-486-3p	Chr. 8p11	Targets the 3′ UTR of BCL11A XL isoform mRNA	Primary human CD34^+^ derived erythroid cell culture	Overexpression of miRNA-486-3p decreases BCL11A protein levels and subsequently increases γ-globin levels. It has modest prediction value to differentiate patients with STEMI from those with stable ischemic heart disease.	[38,39]
miR-190b-5p	chr1: 154193665–154193743	Bind directly to the 3′-UTR of BCL11A	Peripheral blood of 25 pediatric β-thalassemia patients	Markedly upregulated in the peripheral blood of β-thalassemia and shows a significant inverse correlation with BCL11A mRNA levels, while positively correlating with increased HbF.	[40]
miR-6747-3p	chr11: 62567011–62567071	directly binds to the 546–552 nucleotide region of the BCL11A mRNA 3′ UTR	HUDEP-2 and K562	No established association with heart or liver injuries.	[41]
miR-129-5p	Encoded by two distinct genomic loci (miR129-1 chr7: 128207872–128207943, and miR129-2 chr11: 43581394–43581483)	Directly targets BCL11A mRNA by binding its 3′UTR, leading to its downregulation and γ-globin upregulation.	Peripheral blood from β-thalassemia intermediate and major patients, K562, HUDEP-2	Validated as a potential therapeutic and diagnostic biomarker, miR-129-5p is significantly upregulated in β-thalassemia and correlates with higher HbF and better liver function. Might be useful in guiding γ-globin-targeted therapies. Potential diagnostic for ischemic–reperfusion cardiac injury.	[42,66]
miR-17-3p	chr13: 91350605–91350688	Directly targets 3′UTR for BCL11A mRNA	Peripheral blood from β-thalassemia K562	manipulating miR-17-3p in K562 did not alter proliferation, cell cycle, apoptosis, or erythroid differentiation in vitro, suggesting its HbF effect is uncoupled from general erythropoiesis. miR-17-3p is significantly downregulated in peripheral blood exosomes from patients with cardiac ischemic–reperfusion injury.	[43,67]
miRNA-MYB-KLF-BCL11A axis					
miRNA-26B	Chr2: 218402646–218402722	Target the 3′ UTR of MYB mRNA	K562 erythroid cells	Hydroxyurea induces miRNA-26B, which inhibits MYB and ultimately downregulates BCL11A.	[47,48]
miR-15a	Chr. 3: 50049119–50049201	Target the 3′ UTR of MYB mRNA	Primary human CD34^+^ derived erythroid cell culture K562 erythroid cells	miR-15a axis has a role in apoptosis and ferroptosis pathways in the liver, representing a plausible hepatic vulnerability node in β-hemoglobinopathies. Preliminary evidence from liver-injury models implicates miR-15a dysregulation; targeted miR-15a delivery to hepatocytes via liver-tropic nanoparticles warrants investigation as an adjunct to standard SCD/β-thal care.	[14,49,68]
miR-16-1	chr13:50048973–50049061	Target the 3′ UTR of MYB mRNA	Primary human CD34^+^ derived erythroid cell culture K562 erythroid cells	No established association with heart or liver injuries.	[49]
miR-29b-3p	Encoded by two distinct genomic loci (miR29b-1 chr7: 130877459–130877539) and miR29b-2 chr1: 207802443–207802523	Target the 3′ UTR of MYB mRNA	Primary human CD34^+^ derived erythroid cell culture KU812 Leukemia cells Townes SCD mouse model	Evidence that it also inhibits DNMT3A.	[50,51]
miR-326	Chr11: 75335092–75335186	Target the 3′ UTR of KLF1 mRNA	Primary human CD34^+^ derived erythroid cell culture K562 erythroid cells Reticulocytes obtained from β-thalassemia major patients	In primary CD34^+^ progenitors, miR-326 reduced KLF1 protein and BCL11A mRNA but did not significantly change γ- or β-globin transcripts; however, it reduced erythroid maturation (lower glycophorin-A), indicating context-dependent effects.	[24]
miRNA-SOX6-BCL11A axis					
miR-365-3p	Encoded by two distinct genomic loci MIR365A: chr16: 14309285–14309371 and MIR365B: chr17: 31575411–31575521	targets the 3′ UTR of SOX6 mRNA	HUDEP-2 erythroid cells K562 erythroid cells Mouse fetal liver model	BCL11A represses miR-365-3p expression by binding to its regulatory sites. Stable SOX6 knockdown produces a rise in γ- and ε-globin in HUDEP-2 erythroid cells.	[57]
miR-19b-3p	Encoded by two distinct genomic loci MIR19B1: chr13: 91351192–91351278 MIR19B2: chrX: 134169671–134169766	targets the 3′ UTR of SOX6 mRNA	β-thalassemia patients K562 erythroid cells	Its level increased in β-thalassemia patients.	[58]
miRNA-SP1/KLF3 axis					
miR-23a	Chr19: 13836587–13836659	directly repressing the transcriptional repressors SP1 and KLF3	Primary human CD34^+^ derived erythroid cell culture K562 erythroid cells	In primary CD34^+^ erythroid cultures, the dominant effect is β-globin induction.	[60]
miR-27a	Chr19: 13836440–13836517	directly repressing the transcriptional repressors SP1 and KLF3	Primary human CD34^+^ derived erythroid cell culture K562 erythroid cells	In primary CD34^+^ erythroid cultures, the dominant effect is β-globin induction.	[60]

**Table 2:** Nanoparticle systems targeting HSPCs and erythroid progenitors.

Target Cell/Type	Target Marker/Ligand	Nanoparticle Platform	Payload (Targets/Use)	Model	Key Outcome	Specificity	References
HSPCs (including LT-HSCs)	CD117 (c-Kit)	Ionizable LNP (ALC-0315; Ab via PEG-maleimide)	siRNA (CD45); mRNA (Cre, luciferase)	Mouse (Ai14) in vivo; EML in vitro	Cre mRNA: 14 wk. multilineage marking: Myeloid = 90%, B-cell = 70%, T-cell = 50%, RBC = 100% Dose effect: =90% HSPC/LT-HSC at 1 mg/kg; and 25% untargeted. siCD45: =40% KD at 1 mg/kg.	Targeting via CD117 outperformed isotype and other markers (CD49d, CD44, IL-6R); unconjugated LNP gave ~25% editing vs. ~90% with anti-CD117 at 1 mg/kg	[79]
Fetal HSPCs (Lin−/Sca1+/c-Kit+) incl. LT-HSCs; hematopoietic progeny	CD45—F(ab’)_2_ *	Ionizable LNP (C14-490/DOPE/Chol/PEG; “B5/STEM”)	mRNA (GFP, mCherry, Cre); CRISPR (Cas9+sgRNA: GFP, Ttr)	In utero mouse (E13.5); human CD34+ ex vivo	60 h: 30% HSCs vs. 4% untargeted. 4 months: 19% BM HSCs GFP+ vs. 4% untargeted	CD45-dependent: isotype control inactive; competition with free CD45 Ab reduces effect; HepG2 (CD45−) not transfected; adult BM HSCs not transfected	[82]
Bone marrow niche (leukemic) and leukemia cells (ICAM-1+); BM-homing via HSPC cues	BM niche: CD44—hyaluronic acid and Leukemia cells: ITGB2—ICAM-1	HSPC-membrane-coated liposomes (biomimetic)	Cytarabine (Ara-C); ICG tracer for imaging	Mouse leukemia; C1498/Ka539; human AML (ICAM-1)	BM homing ↑ vs. other membranes; BM accumulation; ↑ Disease effect: ↓ leukemia cells/LSCs in BM/spleen/PB; survival ↑.	Mechanism-based: CD44 knockdown ↓ BM targeting; ITGB2 knockdown ↓ leukemia binding; BM HA elevated; co-localization with HA and ICAM-1	[83]
HSPCs (murine Lin−/LSK/MPP/HSC subsets); human CD34+ HSPCs (in vitro)	CD105 (endoglin) antibody	Layer-by-layer liposomes (liposome → PLR → PAA)	None (Cy5-labeled tracking); platform study for targeting	Mouse in vivo (1.5 h); human CD34^+^ ex vivo; human B-cell in vitro	In vivo HSPC association: Mk/E-MPP = 8.5%, HSC = 3.0% (CD105 best). Off-target: PAA outer layer ↓ myeloid uptake.	Anti-CD105 > anti-cKit/anti-CD90/anti-CD45/anti-CXCR4 for murine HSPCs in vivo; anti-CD105 also highest in human CD34+ in vitro	[84]
Human CD34+ HSPCs (ex vivo)	None (ex vivo CD34^+^)	Non-viral RNA-LNP (ex vivo) (NanoAssemblr NxGen)	CRISPR (Cas9+sgRNA: CD33, CD45)	Human CD34+ ex vivo (PB/CB)	KO: 84 ± 6% (CD33), 81 ± 2% (CD45); ~95% viability; >90% proliferation vs. untreated; CFU lineage formation unchanged; scalable manufacturing	Ex vivo gene-specific editing; no in vivo targeting	[85]

* Mouse CD45.2 in vivo; human BC8 ex vivo. Delivery route is intravenous (IV) unless explicitly noted as ex vivo. PB = peripheral blood. CB = core blood. KD = knockdown. MPP = multipotent progenitor. Mk/E-MPP = megakaryocyte/erythroid-biased MPP. PAA = poly(acrylic acid), ↓ =decrease, ↑ = increase.

## Data Availability

No new data were created or analyzed in this study. Data sharing is not applicable to this article.

## Abbreviations

The following abbreviations are used in this manuscript:

3′-UTR	3′ untranslated region
ACS	acute coronary syndrome
AGO2	Argonaute-2
ALT	alanine aminotransferase
AST	aspartate aminotransferase
AUC	area under the ROC curve
BCL11A	B-cell CLL/lymphoma 11A
BM	bone marrow
CB	cord blood
CD34^+^	cluster of differentiation 34–positive (hematopoietic progenitors)
CD71	transferrin receptor 1
CD235a	glycophorin A
CRISPR	clustered regularly interspaced short palindromic repeats (genome editing)
DGCR8	DiGeorge critical region 8
DNMT	DNA methyltransferase
DNMT3A	DNA methyltransferase 3A
Glu6Val	glutamic-acid-to-valine substitution at β-globin position 6
HAT	histone acetyltransferase
HbA	adult hemoglobin (α_2_β_2_)
HbF	fetal hemoglobin (α_2_γ_2_)
HbS	sickle hemoglobin
HBA/HBB	α-/β-globin genes
HDAC1	histone deacetylase 1
HMG	high-mobility group (as in HMG-box of SOX6)
HUDEP-2	human erythroid progenitor cell line
I/R	ischemia–reperfusion
K562/KU812	human erythroleukemia/basophilic leukemia cell lines
KLF1/KLF3	Krüppel-like factor 1/3
LCR	locus control region (β-globin locus)
LNP	lipid nanoparticle
LSC	leukemia stem cell
mRNA	messenger RNA
miRNA	microRNA
MYB	MYB proto-oncogene
NO	nitric oxide
ORF	open reading frame
PB	peripheral blood
PCI	percutaneous coronary intervention
P-/E-selectin	platelet/endothelial selectin
pre-miRNA	precursor microRNA
pri-miRNA	primary microRNA transcript
RBC	red blood cell
RISC	RNA-induced silencing complex
ROS	reactive oxygen species
SCA	sickle cell anemia
SCD	sickle cell disease
sgRNA	single-guide RNA
SOX6	SRY-box transcription factor 6
SP1	specificity protein 1
STEMI	ST-segment elevation myocardial infarction
VCAM-1	vascular cell adhesion molecule-1
ZNF410	zinc finger protein 410
